# An artificial neural network approach for the language learning model

**DOI:** 10.1038/s41598-023-50219-9

**Published:** 2023-12-20

**Authors:** Zulqurnain Sabir, Salem Ben Said, Qasem Al-Mdallal

**Affiliations:** 1 Department of Computer Science and Mathematics, Lebanese American University, Beirut, Lebanon; 2 Department of Mathematical Sciences, College of Science, United Arab Emirates University, P. O. Box 15551, Al Ain, United Arab Emirates

**Keywords:** Engineering, Mathematics and computing

## Abstract

The current study provides the numerical solutions of the language-based model through the artificial intelligence (AI) procedure based on the scale conjugate gradient neural network (SCJGNN). The mathematical learning language differential model is characterized into three classes, named as unknown, familiar, and mastered. A dataset is generalized by using the performance of the Adam scheme, which is used to reduce to mean square error. The AI based SCJGNN procedure works by taking the data with the ratio of testing (12%), validation (13%), and training (75%). An activation log-sigmoid function, twelve numbers of neurons, SCJG optimization, hidden and output layers are presented in this stochastic computing work for solving the learning language model. The correctness of AI based SCJGNN is noted through the overlapping of the results along with the small calculated absolute error that are around 10^–06^ to 10^–08^ for each class of the model. Moreover, the regression performances for each case of the model is performed as one that shows the perfect model. Additionally, the dependability of AI based SCJGNN is approved using the histogram, and function fitness.

## Introduction

There are various differential equations used to model the variety of applications. In disease systems, the susceptible, infected, and recovered (SIR) model is one of the mathematical forms that provide the ideas of different viruses^1,2^. In the SIR model, the addition of exposed, quarantine and treatment classes make these models, SEIR, SIQR and SITR^3–7^. Several differential equations have been used in the modeling of economic and environment systems, food chain networks, ocean engineering and fluid dynamical models^8–13^. The differential form of the models is not only limited to the biological frameworks, but it also has some applications in the language learning progression. The language learning differential models are employed to clarify and describe how an individual would eventually acquire a new language. These models take into account how numerous factors, including motivation, linguistic ability, and exposure to the language, may interact to affect the language learning.

Language acquisition is a complex topic, and no single model can account for all variables that influence it. The fundamental idea is to separate each language’s learning knowledge into three categories: unfamiliar, familiar, and mastered. These categories include vocabulary, pronunciation, listening comprehension, and grammar^14,15^. The unnamed class refers to characteristics of the language that have essentially fallen by the wayside or have not yet been addressed. The term "familiar" refers to language features that are recognized but not fully mastered. “Mastered" (M) specifies the class, which was used to fully learn and is available in a person's working memory. The mathematical learning language differential model is based on three classes, unknown, familiar, and mastered is given as:

1 $$\left\{ \begin{array}{ll} \frac{du(x)}{{dx}} = \frac{\beta f(x)}{{1 + 2\alpha }} - \alpha u(x),&\quad (0) = i_{1} \hfill \\ \frac{df(x)}{{dx}} = - \alpha f(x) - \frac{\beta f(x)}{{1 + 2\alpha }} + \alpha u(x) + \frac{\beta m(x)}{{2 + 4\alpha }},&\quad f(0) = i_{2} , \hfill \\ \frac{dm(x)}{{dx}} = \alpha f(x) - \frac{\beta m(x)}{{2 + 4\alpha }},&\quad m(0) = i_{3} . \hfill \\ \end{array} \right.$$

The sum of *u*, *f* and *m* must be taken as one as they characterize the language’s proportion. $\alpha$ and $\beta$ are the constants that are taken in 0 and 1. The parameter $\alpha$ shows that how rapidly an individual learns the new information, while $\beta$ represents how the person quickly loses or forgets proficiency in information that they already educated.

There are various language systems, e.g., GloVe, Word2Vec, and embedding learned in the process of neural networks defines the words as a vector of high-dimension. The dimensionality reduction schemes can also be used to imagine these embedding in double or triple sizes. The language system provides the attention appliances to examine the weights, which can deliver understandings into those input parts of the model by focusing the predictions. To introduce the combative instances is a significant step in order to assess the robustness of the model. Few examples are provided based on the samples of the input deliberately, which are calculated to misinform the system while performing the vague to a human viewer. To evaluate the performance of the language model based on the examples, which support classify potential susceptibilities, limitations and biases. To integrate the adversarial challenging into the assessment of language model are generation of adversarial text, transferability testing, evaluation of the matrices, fairness and bias examination, iterative testing, adversarial training, tactics of adversarial defense, and human evaluation.

Artificial intelligence (AI) highly depends on language models to enable robots to comprehend and produce human language^16^. These systems are a type of machine learning that uses the statistical patterns to predict the probability of the next sequence of words in each text or sentence^17^. Language models can recognize and learn patterns that how people use language, like context, syntax, and semantics. This enables language models to carry out a variety of tasks involving language processing, e.g., text categorization, sentiment analysis, chatbot generation, translation, and summarization^18,19^. Moreover, language systems are a crucial part of neural translation models that involve the process of deep learning to transform text across different languages^20–27^.

The current investigations present the solutions of learning language differential model by using the artificial intelligence (AI) procedure of scale conjugate gradient neural network (SCJGNN). In recent years, the stochastic computing solvers have been exploited in various applications, like susceptible, exposed, infected and recovered nonlinear model based on the worms propagation using the networks of wireless sensor^28^, eye surgery differential system^29,30^, predictive networks for the delayed model of tumor-immune^31^, HIV infection system^32,33^, delay differential model based avian influenza^34^, Lane-Emden model^35,36^, influenza-A epidemic system^37^, smoking model^38,39^, multi-delayed model based tumor oncolytic virotherapy^40^, thermal explosion model^41^, and biological differential system^42^. Some novel features of this research are presented as:

- The solutions of the learning language differential model using the stochastic AI along with the SCJGNN solver are presented successfully.
- The competence of Adams numerical scheme is confirmed to accomplish the dataset using the certification and train/test data process.
- The constant and reducible absolute error (AE) validate the implication of SCJGNN method to solve the learning language differential model.
- The reliable matching of results, a strong treaty using the typical manufactures provide the precision of AI along with the SCJGNN for solving the language model.

The remaining paper’s portions are given as: "Designed stochastic AI along with the SCJGNN solver" is related to the designed computing solver. "Numerical results of the language model" designates the output representations; the conclusions are provided in the final section.

## Designed stochastic AI along with the SCJGNN solver

**Figure 1:**
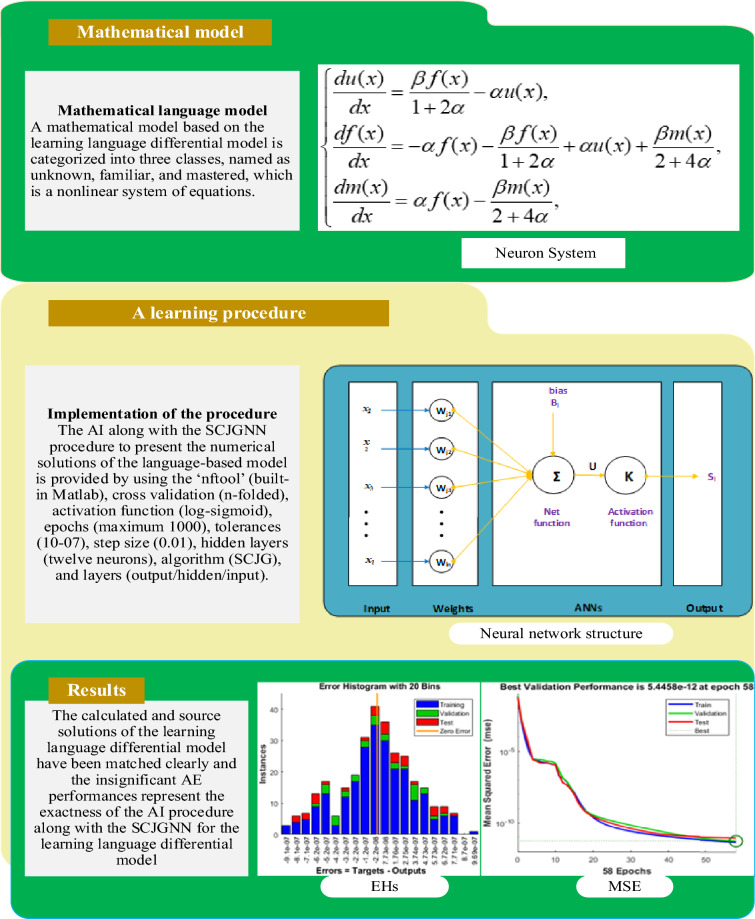
Graphical representations for the SCJGNN solver to solve the learning language differential model.

**Figure 2:**
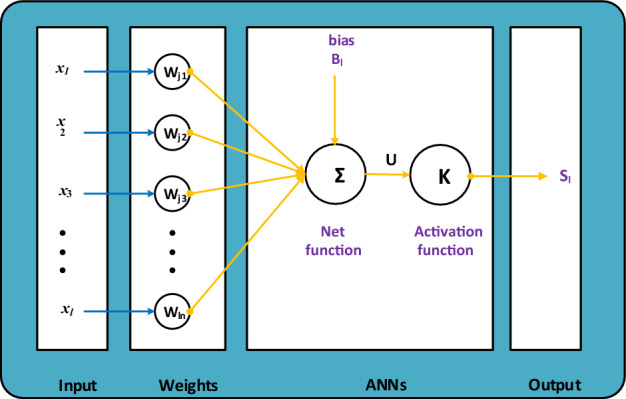
A layer structure of the neural network.

This section portrays the computational framework using the AI along with the SCJGNN in two steps. The SCJGNN features are stated together with the execution process in Fig. 1, however the network performances based on the multi-layers are depicted in Fig. 2. The AI along with the SCJGNN procedure for the language-based model is provided by using the ‘nftool’ (built-in Matlab), cross validation (*n*-folded), activation function (log-sigmoid), epochs (maximum 1000), tolerances (10^–07^), step size (0.01), hidden layers (twelve neurons), algorithm (SCJG), and layers (output/hidden/input). The label statics using the performance of training with input/target are obtained through the basic outputs.

**Figure 3:**
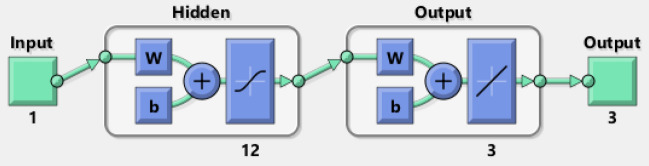
Multi-layers construction for the DDMTV.

The neural network constructions involve various major forms, e.g., architecture defining, stipulating the cost function, selecting the activation function, and training of the model. To integrate the scaling into this procedure might mean considering in order to deal with the input topographies, regularization, and weight initialization. Some of the steps of the general structure for neural network designing with a motivation on scaling are presented as:

- Problem description: The problem is define based on the language learning model using the process of regression by selecting the nature of data and the value ranges for input topographies.
- Data preprocessing: Standardize or normalize the input topographies to an analogous gage. The data preprocessing provides the support to the model in order to converge quicker during the process of training in order to upgrade the performance.
- Design of architecture: The design of neural network based on the types and number of layers is presented by using the layers of normalization to support the interior covariate shift that is relevant to scaling.
- Weight initialization: Select an appropriate scheme to initialize the weights. The weights have been selected with care and concentrations.
- Activation functions: The activation function is also selected carefully, the log-sigmoid function is selected in this study, which is shown in the Fig. 3 in the process of hidden layers.
- Loss function: This function is selected by using the mean squared error (MSE) for regression.
- Optimization: The optimization is performed by using the SCJG.
- Training: The training of the model is performed in order to train the data, and to observe the performance using the sets of authentication.
- Evaluation: The evaluation of the model is performed based on the set of separate tests to measure its generalization presentation.

Table 1 presents the adjustment of the parameters by using the SCJGNN to solve the language nonlinear mathematical model. To assess the limitations and advantages of one of the numerical schemes SCJGNN, the following factors are considered, like the performance of the method is used to approximate the problem’s result, the method perform the consistently and quickly convergence for the solutions, the SCJGNN scheme perform computationally competent, particularly large-scale of problems, the robustness of the SCJGNN scheme is performed to deal with numerous forms of the input data.

**Table 1:** Adjustment of the parameters based on the SCJGNN.

Index	Settings
Minimum gradient	10^–08^
Fitness goal (MSE)	0
Activation function	Log-sigmoid
Maximum Mu	10^10^
Maximum Epochs	1000
Hidden neurons	12
Output layer	Single
Validation statics	13%
Increasing Mu performances	9
Sample collection	Arbitrary
Lower Mu values	0.1
Authentication fail amount	6
Training data	75%
Input layer	Single
Testing data	12%
Adaptive parameter	7 × 10^–05^
Dataset	Adam technique
Stoppage standards	Default
Adam solver executions	Default

The computational stochastic AI along with the SCJGNN solver performances has been presented to solve the learning language differential model using the twelve neurons, which is validated with the optimal performance of underfitting/overfitting using the process of train and corroboration at epochs 49, 58 and 55. The under fitting (premature) convergence is provided by taking small values of the neurons, while the comparable accuracy via the higher complexity (overfitting) is monitored for larger values of the neurons. The label data based on the training and input/target measures is performed through the basic outputs with testing (12%), validation (13%), and training (75%) ratio for solving the nonlinear learning language differential model. The predictable values are found in input [0, 1] through the computational stochastic AI along with the SCJGNN solver are presented to solve the learning language differential model. The layers performances have been performed in Fig. 3 for solving the learning language differential model.

## Numerical results of the language model

The current section performs the solutions of the learning language differential system by using the computing framework of the AI along with the SCJGNN solver. Three different cases of the model are presented as:

*Case 1*: Consider the values $\alpha = 0.5$, and $\beta = 0.1$ are in Eq. (1) as:

2 $$\left\{ \begin{array}{ll} \frac{du(x)}{{dx}} = \frac{1}{20}f(x) - \frac{1}{2}u(x),&\quad (0) \,=\, 1, \hfill \\ \frac{df(x)}{{dx}} = - \frac{11}{{20}}f(x) + \frac{1}{2}u(x) + \frac{1}{40}m(x),&\quad f(0) \,=\, 0, \hfill \\ \frac{dm(x)}{{dx}} = \frac{1}{2}f(x) - \frac{1}{40}m(x),&\quad m(0) \,=\, 0. \hfill \\ \end{array} \right.$$

*Case 2*: Consider the values $\alpha = 0.5$, and $\beta = 0.5$ are in Eq. (1) as:

3 $$\left\{ \begin{array}{ll} \frac{du(x)}{{dx}} = \frac{1}{4}f(x) - \frac{1}{2}u(x),&\quad u(0) \,=\, 1, \hfill \\ \frac{df(x)}{{dx}} = - \frac{3}{4}f(x) + \frac{1}{2}u(x) + \frac{1}{8}m(x),&\quad f(0) \,=\, 0, \hfill \\ \frac{dm(x)}{{dx}} = \frac{1}{2}f(x) - \frac{1}{8}m(x),&\quad m(0) \,=\, 0. \hfill \\ \end{array} \right.$$

*Case 3*: Consider the values $\alpha = 0.5$, and $\beta = 0.9$ are in Eq. (1) as:

4 $$\left\{ \begin{array}{ll} \frac{du(x)}{{dx}} = \frac{9}{20}f(x) - \frac{1}{2}u(x),&\quad u(0) \,= \,1, \hfill \\ \frac{df(x)}{{dx}} = - \frac{19}{{20}}f(x) + \frac{1}{2}u(x) + \frac{9}{40}m(x),&\quad f(0) \,=\, 0, \hfill \\ \frac{dm(x)}{{dx}} = \frac{1}{2}f(x) - \frac{9}{40}m(x),&\quad m(0) \,=\, 0. \hfill \\ \end{array} \right.$$

**Figure 4:**
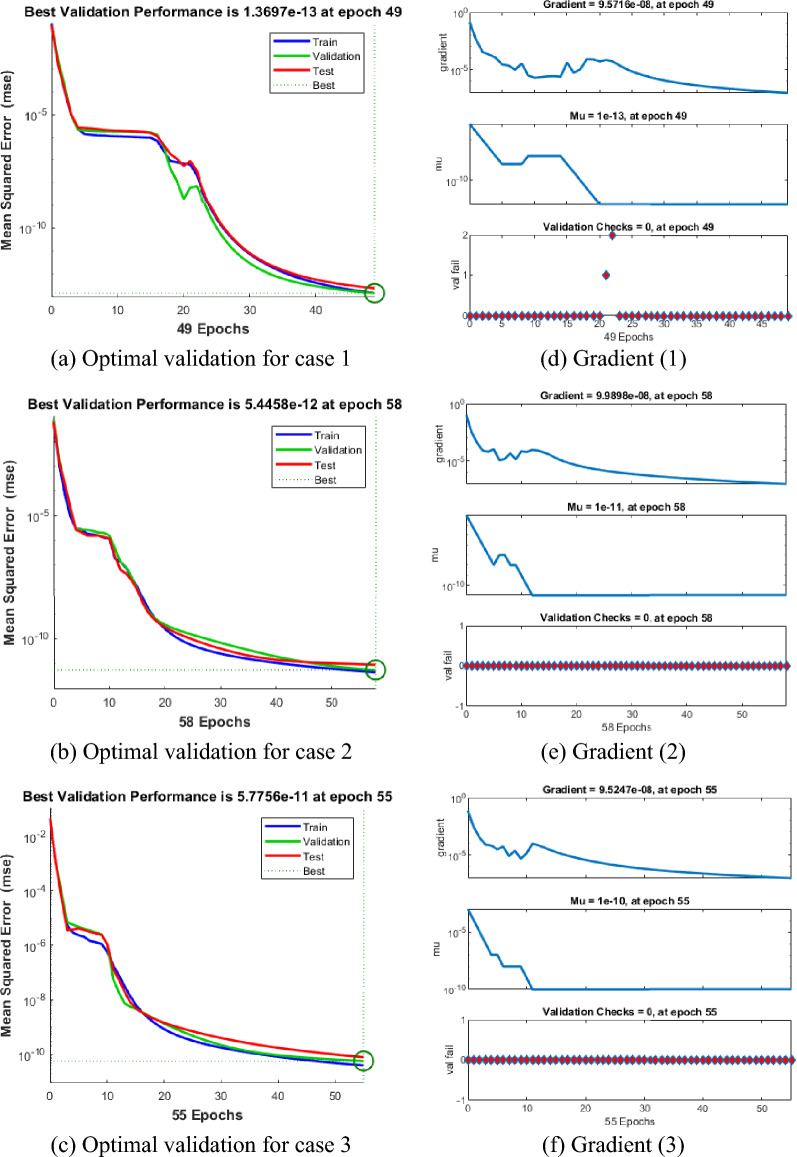
Optimal verification and gradient for the learning language differential model.

**Figure 5:**
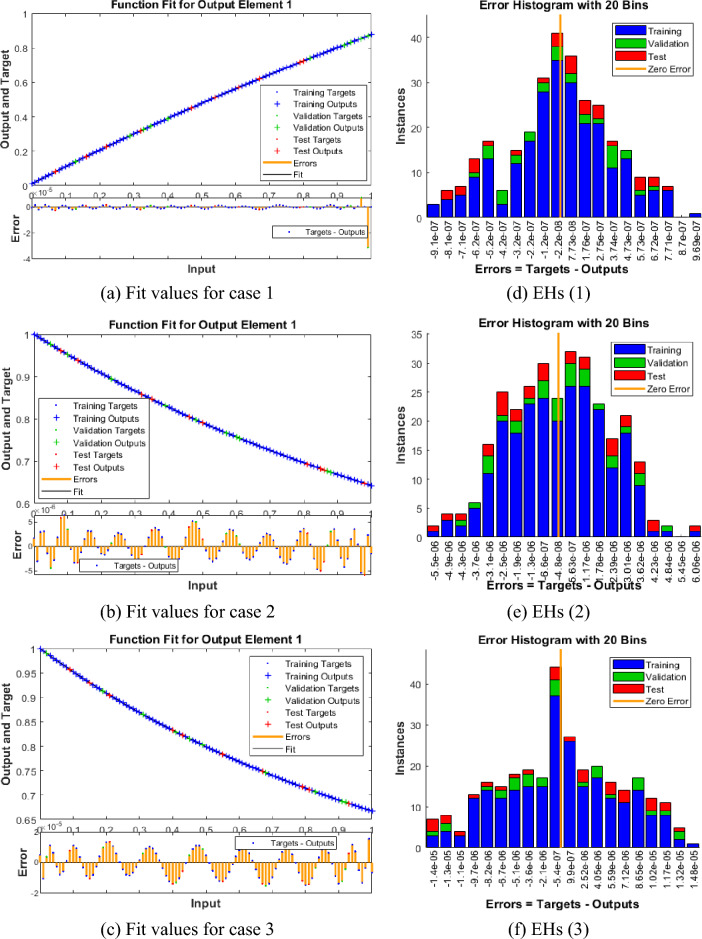
Fit and EHs performances for the learning language differential model.

**Figure 6:**
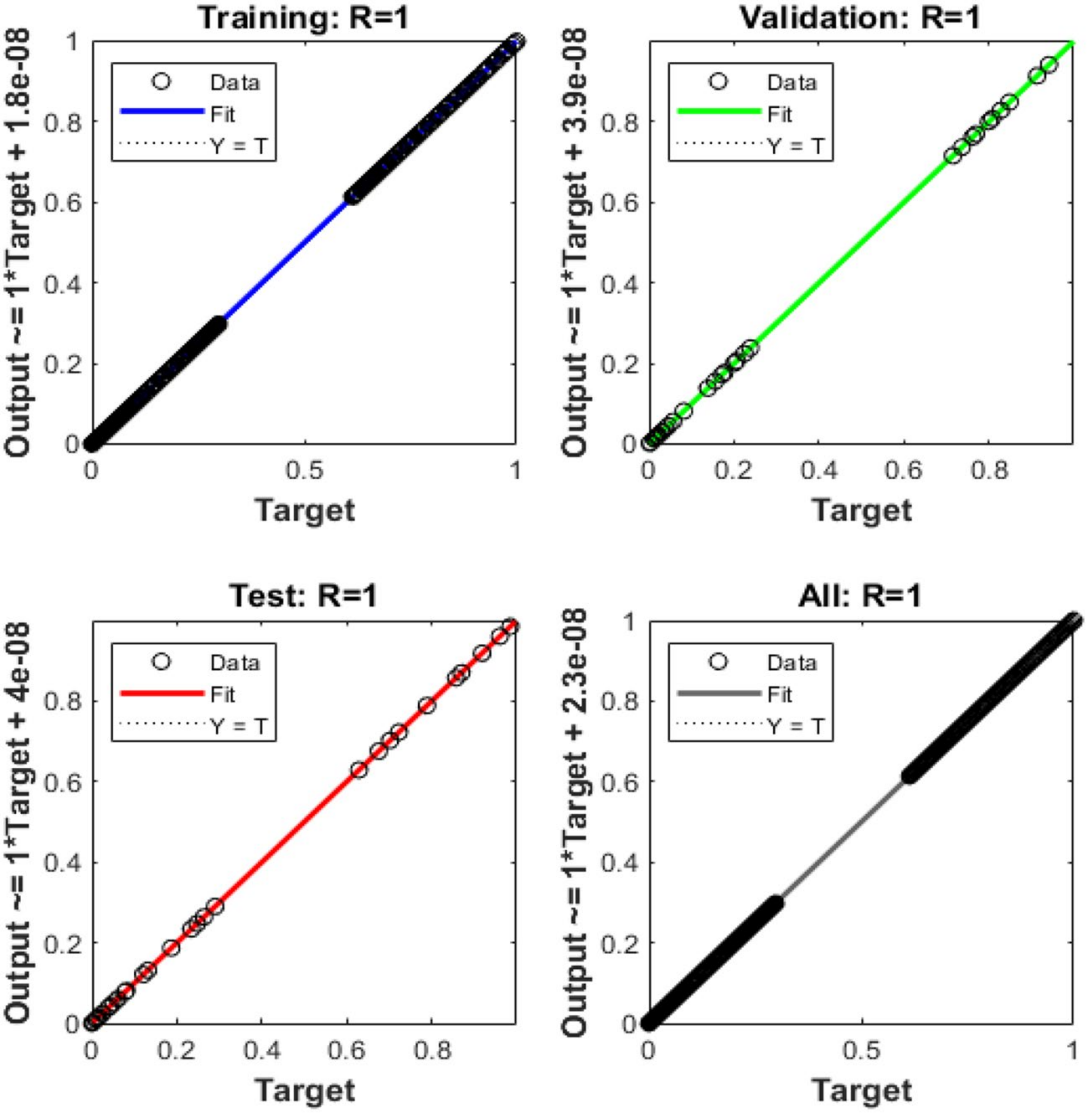
Case 1: regression for the learning language differential model.

**Figure 7:**
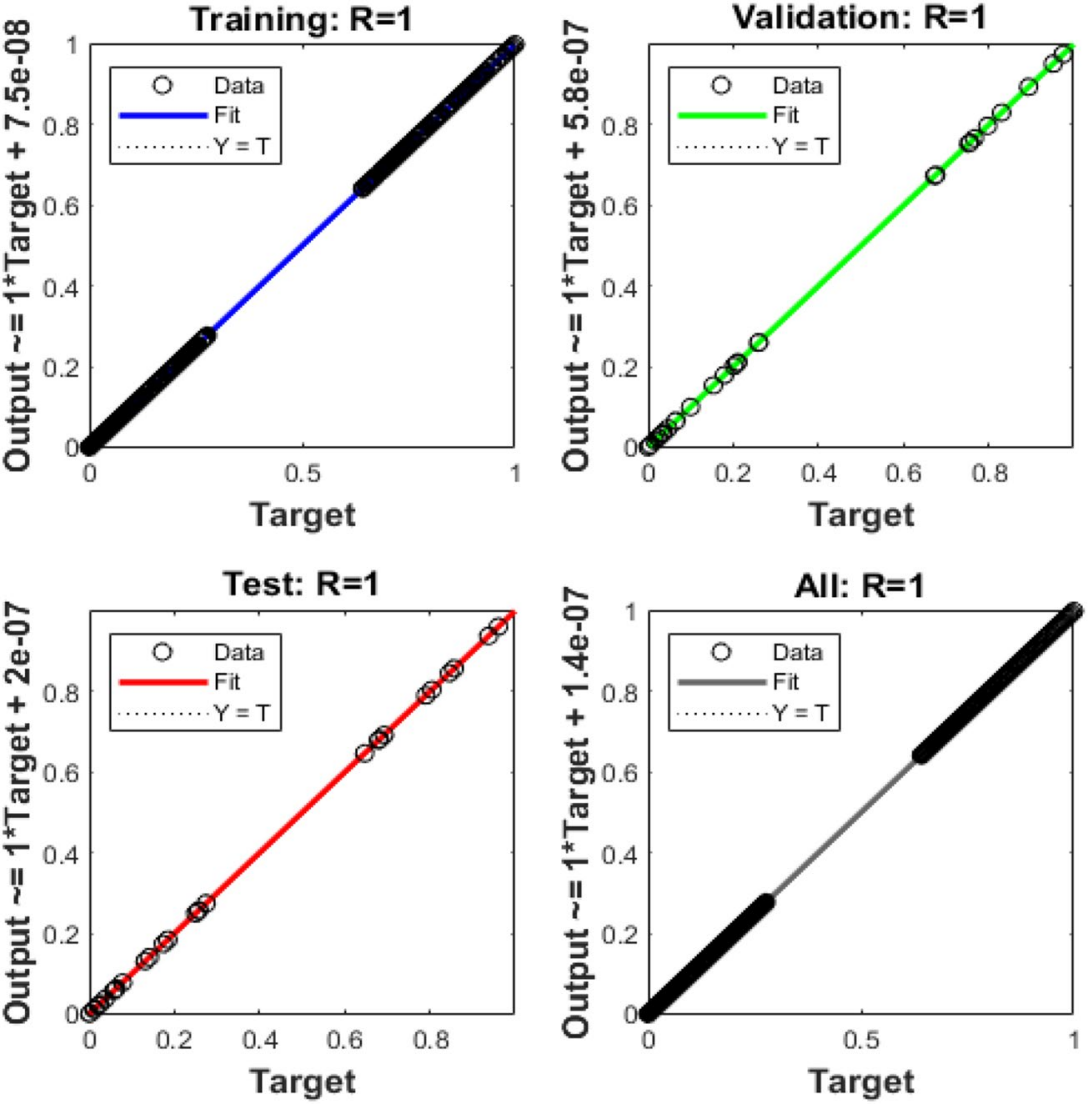
Case 2: regression for the learning language differential model.

**Figure 8:**
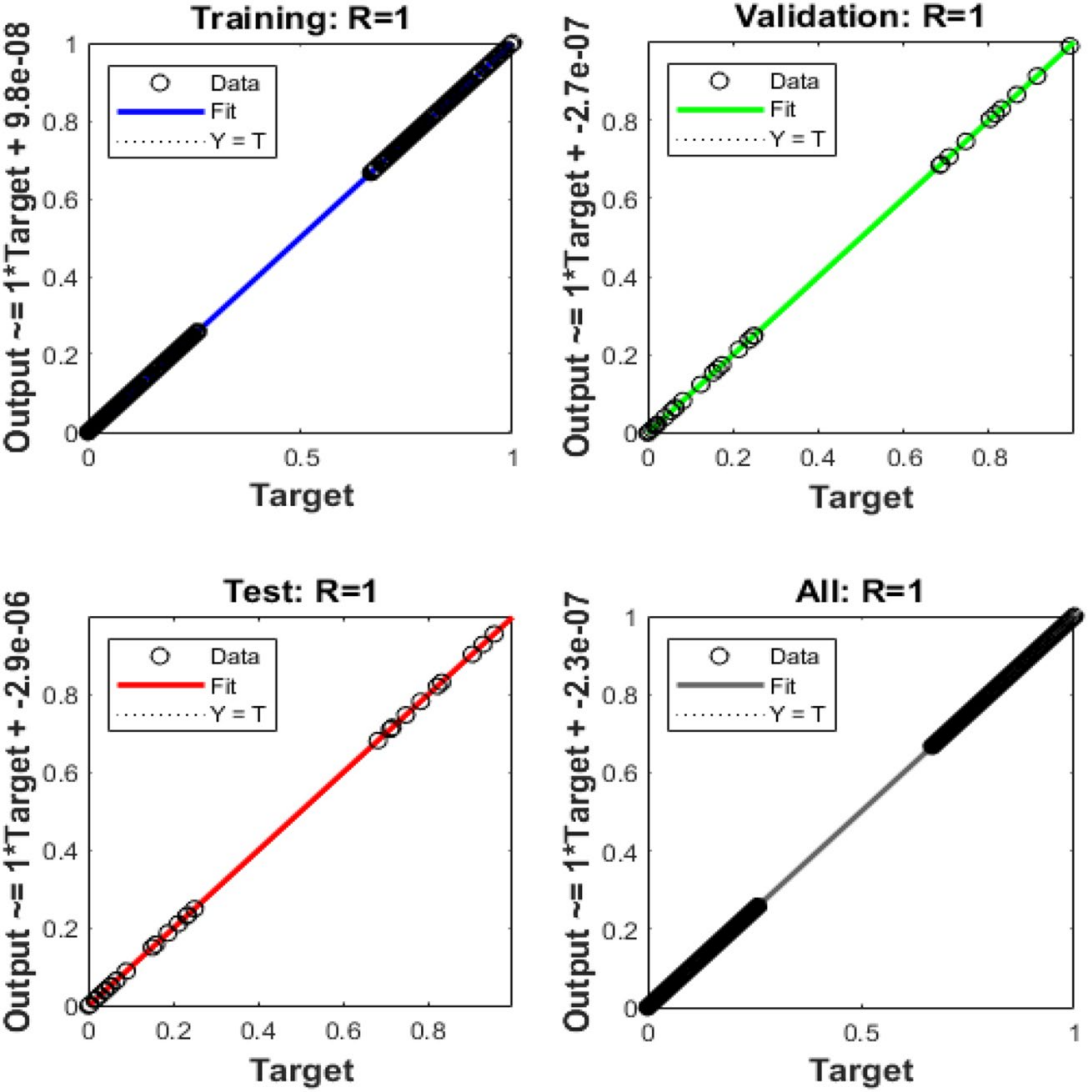
Case 3: regression for the learning language differential model.

Figures 4, 5, 6, 7 and 8 represent the AI procedure along with the SCJGNN for the learning language differential model. Figure 4 presents the MSE and state of transition (SoT) by applying the AI procedure along with the SCJGNN. The assessed MSE performances have been illustrated in Figs. 4a–c based on the best curves, training, and accreditation, while the values of the SoT are given in Fig. 4d–f. The optimal outputs of the learning language differential model are represented at epochs 49, 58 and 55, which are shown as 1.36973934 × 10^–13^, 5.44578 × 10^–12^ and 5.77556 × 10^–11^. The gradient for the classes unknown, familiar, and mastered are measured as 9.5716 × 10^–08^, 9.9898 × 10^–08^ and 9.5247 × 10^–08^. These gradient curves indicate the convergence, exactitude, and exactness, and of the AI along with the SCJGNN solver for the nonlinear learning language differential model. Figure 5 depicts the fitting curves for the learning language differential model based on the comparison of results. Figure 5d–f signifies the values of the error histogram (EHs) for the learning language differential model through the stochastic performances of AI along with the SCJGNN solver. These measures have been reported as 7.73 × 10^–08^ for unknown class, 5.63 × 10^–07^ for familiar category, and 9.90 × 10^–07^ for mastered class. Figures 6, 7 and 8 presents the correlation graphs for the differential form of the learning language model by applying the stochastic computing AI along with the SCJGNN performance. These graphs show that the determination coefficient *R*^*2*^ is 1 for the unknown, familiar, and mastered categories. The curves based on the testing, validation, and training representations authenticate the precision of the AI along with the SCJGNN for solving the differential form of the learning language model. The convergence of the train, epochs, endorsement, backpropagation, test and intricacy are tabulated in Table 2. The complexity performances using the AI along with the SCJGNN for solving the differential form of the learning language model are capable to prove the network’s training (epoch performance).

**Table 2:** AI along with the SCJGNN solver for the learning language differential model.

Case	MSE			Performance	Mu	Gradient	Epoch	Time
	Test	Authorization	Train					
I	2.256 × 10^–13^	1.369 × 10^–13^	1.393 × 10^–13^	1.39 × 10^–13^	1 × 10^–13^	9.57 × 10^–08^	49	1
II	8.848 × 10^–12^	5.445 × 10^–12^	4.530 × 10^–12^	4.53 × 10^–12^	1 × 10^–11^	9.99 × 10^–08^	58	1
III	7.906 × 10^–11^	5.775 × 10^–11^	4.020 × 10^–11^	4.02 × 10^–11^	1 × 10^–10^	9.52 × 10^–08^	55	1

**Figure 9:**
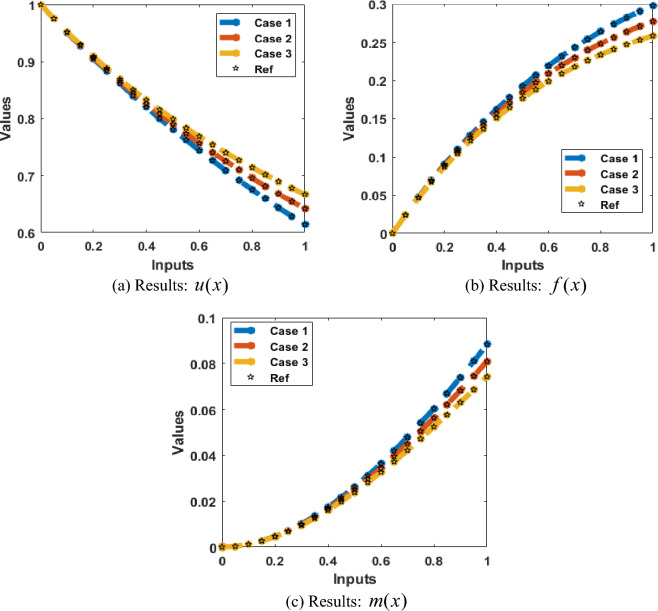
Obtained and reference results for the learning language differential model.

**Figure 10:**
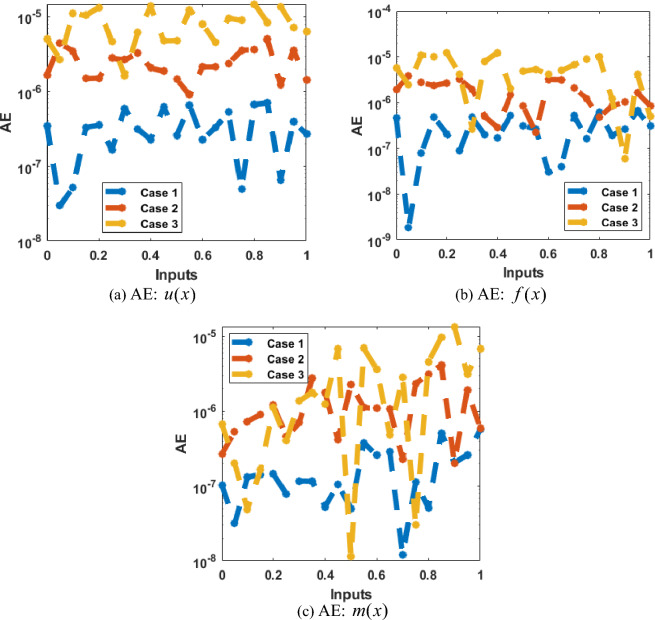
AE for the learning language differential model.

Figures 9 and 10 shows the outputs comparison along with the values of the AE using the obtained and reference solutions for the learning language differential model. The calculated and source solutions of the learning language differential model have been matched clearly. Figure 9 provides the AE measures for each class of the learning language differential model. The values of the AE is a metric, which is applied to assess the accuracy of the language learning model. It provides the average absolute difference that is calculated between the obtained and actual performances. The implication of the AE is based on its interpretability and simplicity. Dissimilar to some other operator like mean absolute deviation, the AE provides equal weights to each error and not used to penalize the large values of the errors more deeply. In case 1, the AE measures for the unknown class are reported as 10^–06^ to 10^–07^, whereas the familiar, and mastered categories are performed as 10^–05^ to 10^–06^. In case 2, the AE are performed as 10^–06^ to 10^–07^ for unknown, 10^–05^ to 10^–07^ for familiar category and 10^–05^ to 10^–06^ for mastered class of the model. In case 3, the AE measures are reported as 10^–07^ to 10^–09^ for unknown, 10^–05^ to 10^–07^ for familiar category and 10^–05^ to 10^–08^ for mastered dynamic of the mathematical model. These insignificant AE performances represent the exactness of the AI procedure along with the SCJGNN for the learning language differential model.

## Conclusions

The motive of current investigations is to present the solutions of the learning language differential model by applying the artificial neural networks. The differential models are not only played a role in the diseased spread modeling, but also have some role in the learning language differential models. Hence a language-based differential model is numerically presented through the process of artificial intelligence along with the optimization of scale conjugate gradient neural network. The mathematical dynamics of the learning language differential model is characterized into three forms, called as unknown, mastered, and familiar. The AI based SCJGNN procedure has been programmatic by applying the statics of testing (12%), validation (13%), and training (75%). In the process of neural network for solving the learning language model, a transfer function log sigmoid, SCJG optimization, twelve numbers of neurons, output, hidden and output layers have been presented in this stochastic computing framework to present the numerical solutions of the learning language model. The correctness of the AI based SCJGNN has been observed through the overlapping of the obtained and source (Adam) results. The negligible absolute error has been performed around 10^–07^ to 10^–09^ for respective cases of the language model. In addition, the reliability of the AI based SCJGNN is observed by applying the function fitness, histogram, and correlation/regression of the language differential model.

In future, the designed AI based SCJGNN structure can be developed for the computational framework of the mathematical model, fluid dynamics, and nonlinear models^43–52^.

## Data Availability

All data generated or analysed during this study are included in this article.
